# A pilot screening for cognitive impairment through voice technology (WAY2AGE)

**DOI:** 10.1186/s40359-023-01212-4

**Published:** 2023-05-23

**Authors:** Carmen Moret-Tatay, Isabel Iborra-Marmolejo, María José Jorques-Infante, Gloria Bernabé-Valero, María José Beneyto-Arrojo, Tatiana Quarti Irigaray

**Affiliations:** 1 Faculty of Psychology, Valencia Catholic University Saint Vincent Martyr (UCV), Burjassot, Valencia, Spain; 2 Department of Psychology, Pontifical Catholic University of Rio Grande do Sul (PUCRS), Rio Grande do Sul, Brazil

**Keywords:** Voice-bot, Cognitive impairment, Well-being, Technology adoption, Elderly

## Abstract

Voice technology has grown exponentially, offering an opportunity to different fields, such as the health area. Considering that language can be a sign of cognitive impairment and most screening tools are based on speech measures, these devices are of interest. The aim of this work was to examine a screening tool for Mild Cognitive Impairment (MCI) through voice technology. For this reason, the WAY2AGE voice Bot was tested across Mini-Mental (MMSE) scores. The main results depict a strong relationship between MMSE and WAY2AGE scores, as well as a good AUC value to discriminate between no cognitive impairment (NCI) and MCI groups. However, a relationship between age and WAY2AGE scores, but not between age and MMSE scores, was found. This would indicate that, even if WAY2AGE seems sensitive to detect MCI, the voice tool is age-sensitive and not as robust as the traditional MMSE scale. Future lines of research should look more deeply into parameters that distinguish developmental changes. As a screening tool, these results are of interest for the health area and for at-risk older adults.

The increase of digital devices in the last decade offers opportunities for different fields of action. While companies have reduced costs and systematized processes through Artificial Intelligence (AI), its application is not alien to other fields, such as healthcare [1]. An increasing social pressure for digital adoption, after Covid-19 outbreak, has offered an opportunity to overcome distancing constraints. Particularly, this was the case for older adults with underlying health problems [2]. However, there has been much discussion regarding differences in the adoption across age groups [3]. Even if digital divide between older and younger adults seems to be narrowed in recent years, different variables, both externals and internals, have been described as modulators of this digital divide [4].

The increasing use of voice assistants (VA) has opened up new possibilities in the healthcare field, particularly for the inclusion of older adults. While some technologies requiring manual dexterity pose challenges for certain individuals [5], VAs have the potential to overcome these obstacles. Furthermore, VAs can support tasks such as triaging, management, and remote monitoring, ultimately reducing healthcare service costs [6]. A systematic review suggests that the future will witness a decrease in the workforce and number of professional caregivers, while the number of older adults will continue to rise [7]. Consequently, innovative technologies are of great interest in the healthcare sector, not only for patients but also for lightening the workload of healthcare workers.

It should be noted that classical approaches to assess cognitive impairment are based on language performance [8]. This is not really surprising, as a conversation requires the correct functioning of different cognitive processes [9]. Thus, the analysis of vocal components seems to be a promising field combining AI and clinical medicine [6, 8], even for degenerative diseases [10–12].

In this context, the implementation of screening tools for cognitive impairment is a topic of interest. Although widely used tools such as the Mini-Mental State Examination or MMSE [13] have telephone adaptations [14–16], to our knowledge, there are no adaptations for the VA. A piece of research in the field proposed a tool in the field based on Azure cognitive services, named WAY2AGE [17]. This is not an adaptation of the MMSE, but a proposal that contains different dimensions described in some of the most wide-spread tools such as MMSE, COWA (Controlled Oral Word Association Test) [18] and F-A-S (a subtest of the Neurosensory Center Comprehensive Examination for Aphasia [19, 20]. The areas under assessment involve mood, temporal and time orientation, spatial orientation, autobiographical memory, verbal fluency, and work memory. However, WAY2AGE does not involve all areas under assessment in tools such as MMSE. By only measuring verbal responses, measures such as writing, or drawing are not included. This limitation has not been examined in previous work. WAY2AGE to date has only assessed the perception of health professionals in clinical assessment. Hence, the main goal of this study is to ascertain the effectiveness of WAY2AGE in correctly categorizing individuals with MCI, as assessed through the MMSE.

## Method

### Participants

A sample of 36 individuals over 60 years of age volunteered to participate in the study. They referred to be previously classified into groups according to a medical assessment and this classification was confirmed using the standard diagnostic criteria regarding MMSE [13] in its Spanish adaptation [21]: (1) no cognitive impairment (NCI) and (2) mild cognitive impairment (MCI). The inclusion criteria were described as follows: (i) Be aged between 60 and 95 years old; (ii) to be a native Spanish speaker and have no hearing impairment; (iii) to demonstrate no substantial interference with normal daily activities as determined by clinical interview; (iv) No dementia diagnosed. Exclusion criteria also included or not being able to read and write, medical or psychiatric conditions, and current self-reported mood status.

A cut-off point (24 points) was applied in relation with previous literature, also considering level of education for the MMSE correction [22]. In terms of education the MCI group was divided into 50% basic studies and 50% without studies. The NCI group refereed a 13.7% without studies, 40.9% basic studies, 40.9% University studies and a 4.5% Postgraduate studies. The NCI group has a mean age = 70.72 (SD = 9.25) while the MCI was 75.42 (SD = 12.11). No statistically significant differences across age were found though Mann-Whitney U test (p > 0.05). With regards to sex, a 45.5% were men in the NCI group, while a 21.4% in the MCI group. The study was carried out in accordance with the Helsinki Declaration. Thus, to participate in the different studies, all participants gave written informed consent (approval from the ethical committee of the Catholic University of Valencia ref: UCV/2020–2021/163). It should be notes that some participants did not have any former education, but all of them know how to right and read, ensuring the ability to consent.

### Materials and procedure

First, a sociodemographic battery of questions and a brief interview was carried out. Secondly, the Mini-Mental State Examination (MMSE) in its Spanish adaptation by Lobo et al. (1999) was employed. Afterwards, the WAY2AGE voice-bot based on an Azure cognitive service [17] was administrated by trained assistants and psychologists. In this way, participants had to answer to a voice bot different question, being encouraged to do it without time limit. WAY2AGE involves the following questions and areas of interest:

*Item 1 (time orientation): what day is it and what day of the week?*

*Item 2 (spatial orientation): where are we now?*

*Item 3(Time orientation): What is the name of the current and previous president of the country?*

*Item 4 (lexical access): list for one minute all the names of animals you know*

*Item 5 (short memory): repeat the following words: Apple, spoon, bicycle, book, and streetlamp*

*Item 6 (Attention/Calculation*): *Count backwards from three-to-three numbers starting from 29*.

*Item 7 (Working memory): Repeat the previous words.*

Each question scored from 0 to 1 according to whether it was incorrect or correct respectively. In the case of number of animals, the total number of correct animals identified without repetitions was divided by 60 (number of seconds of response time). The total sum gives the overall WAY2AGE score.

### Analysis

Analyses were conducted with JASP (Version 0.12.2, Amsterdam, The Netherlands) and SPSS v23 (IBM). Non-parametric approaches were employed to analyse differences across MMSE groups (NCI versus MCI). A logistic regression was employed to predict presence/absence of MCI through the different WAY2Age components, under the Wald test. Lastly, the receiver operating characteristic curve (ROC) analysis was performed to assess the diagnostic accuracy of each parameter at each temporal resolution. The area under the ROC curve (AUC) results were considered excellent for AUC values between 0.9 and 1, good for AUC values between 0.8 and 0.9, as described in previous literature [23].

## Results

**Fig. 1:**
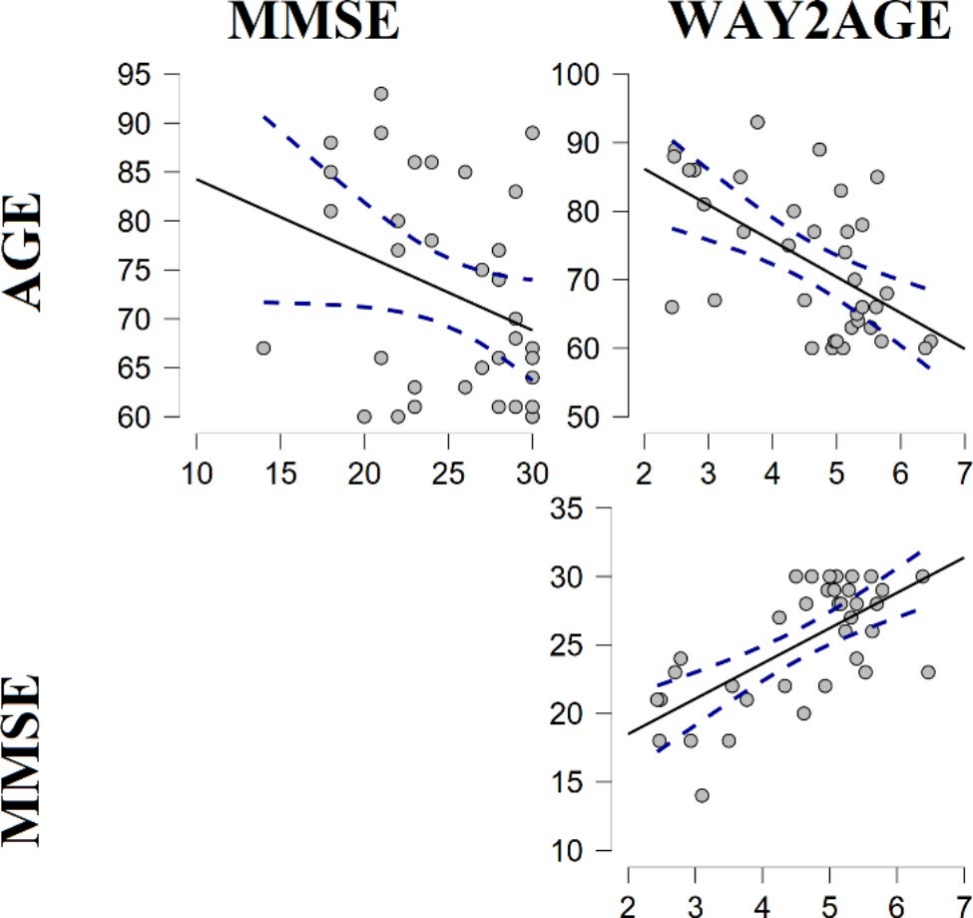
Spearman’s Correlations across Age, MMSE and WAY2AGE scores, including confidence intervals

The MMSE score for the NCI group was 28.18 (SD = 1.87), while the MCI group 20.43 (SD = 2.59). Differences across groups were examined for WAY2AGE total score reaching the statistically significant level through the Mann-Whitney U test: W = 224.50; p < 0.05; Rank Biserial correlation = 0.458; 95% CI [0.10;0.70]. Spearman’s Correlations depicted a direct relationship between MMSE and WAY2AGE scores: rho = 0.576; p < 0.001. Of note, MMSE were not related to Age (p > 0.05), but an inverse relationship was found across Age and WAY2AGE scores (rho=-0.512; p < 0.01). Figure 1 depicts these relationships.

Secondly, a logistic regression was carried out. Two models were carried out. First, the WAY2AGE single scores were entered as the predictors and the outcome variables was the group (MCI versus NCI) based on MMSE scores. Age was also included as a predictor. The first model suggested a statistically significant relationship (χ^2^(35) = 27.06, p < 0.001). McFadden’s indicated a good model fit, R^2^ = 0.56. Secondly, WAY2AGE total scores were considered as a single predictor in model 2. The second model suggested a statistically significant relationship (χ^2^(35) = 14.20, p < 0.001). McFadden’s indicated a good model fit, R^2^ = 0.29. Table 1 indicates the coefficients for the Wald test.

**Table 1:** Logistic Regression for the WAY2AGE items in the prediction of group (MCI versus NCI) in Model 1, as well as the overall score of WAY2AGE in the prediction of group (Model 2)

					Wald Test	
		Estimate	Standard Error	*z*	Wald Statistic	
Model 1	(Intercept)	40.632	3.956.202	0.010	1.055e -4	0.992
	Item 1	-4.806	2.414	-1.991	3.963	0.047
	Item 2	-17.212	3.956.181	-0.004	1.893e -5	0.997
	Item 3	-0.812	2.183	-0.372	0.138	0.710
	Item 4	4.739	6.298	0.752	0.566	0.452
	Item 5	-13.663	6.418	-2.129	4.532	0.033
	Item 6	-5.704	2.783	-2.050	4.201	0.040
	Item 7	3.990	2.500	1.596	2.548	0.110
	Age	-0.085	0.106	-0.801	0.641	0.423
Model 2	(Intercept)	10,765	5981	1800	3240	0.072
	WAY2AGE	-1653	0.601	-2753	7578	0.006
	Age	-0.051	0.053	-0.968	0.938	0.333

**Fig. 2:**
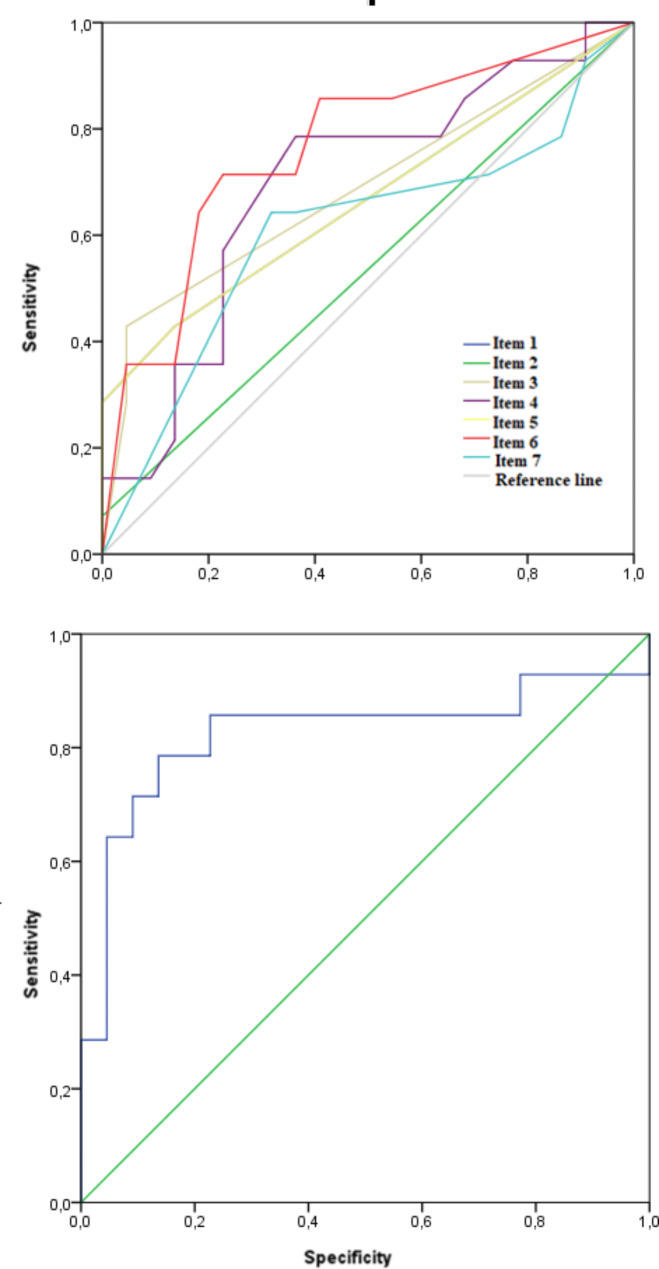
Sensititvity and Specitivity for each WAY2AGE item (top) and total scores (bottom)

Lastly, the area under the ROC curve (AUC) was examine twice: for each WAY2AGE item and for WAY2AGE total scores. For the model on each WAY2AGE item, the highestAUC value was 0.77. Nevertheless, the sensitivity was 0.79 while specificity 0.90. In relation to WAY2AGE total score, the results were considered good (AUC = 0.825; SE = 0.08; p < 0.01; 95% IC [0.65;0.99]). In this case, the sensitivity was 0.64 while specificity 0.95. Figure 2 depicts Specificity and Sensitivity while Table 2 AUC according each WAY2AGE component and total scores.

**Table 2:** AUC for each WAY2AGE component and total scores

Variables	AUC	SE	p	95% asymptotic confidence interval	
				Lowe Limit	Upper limit
Item 1	0.666	0.099	0.098	0.472	0.860
Item 2	0.536	0.101	0.721	0.337	0.734
Item 3	0.688	0.097	0.060	0.498	0.879
Item 4	0.696	0.092	0.050	0.516	0.877
Item 5	0.666	0.099	0.098	0.472	0.860
Item 6	0.766	0.084	0.008	0.601	0.931
Item 7	0.607	0.104	0.284	0.403	0.811
WAY2AGE total scores	0.825	0.086	0.001	0.657	0.993

*SE = Standard Error

## Discussion and conclusions

The aim of this work was to examine a screening tool for MCI through voice technology. In simpler terms, the study aimed to determine if WAY2AGE is capable of accurately classifying MCI as evaluated by the MMSE. For this reason, the WAY2AGE voicebot was tested across MMSE scores. This bot has been examined in terms of usability for healthcare professionals in previous literature [17], but not directly tested with the final target: older adults The main results depict a strong relationship between MMSE and WAY2AGE scores, as well as a good AUC value to discriminate between NCI and MCI groups.

It should be noted that most screening tools are based on language assessment, and according to literature, language can be used as an early marker of cognitive decline [10]. In this way, it is expected that speech characteristics reflect early cognitive changes in at-risk older adults. However, a large inter-rater variability has been described between clinicians, not providing an universally accepted system to describe language impairment yet [24]. In this scenario, VA can be an emerging ground-breaking tool, providing volume of data in this front. In addition, VAs might favour the adoption of digital technology in older people, as they do not have to rely on manual resources.

WAY2AGE was based on items of different cognitive components measured verbally. The item that depicted the best AUC was the calculation/attention item number 4, based on the Brown-Peterson interference paradigm [25]. Nevertheless, one should bear in mind that the total computation of items produced the best results, in other words, the WAY2AGE total score. This would point to the need for a multidimensional assessment of cognitive performance [26]. WAY2AGE is based on verbal responses, but only included a lexical access task. Future lines of research should examine factors underlying the language itself that offer the potential of this test. For this purpose, the use of Natural Language Processing is suggested. This would allow, for example, to examine aspects such as lexical networks in item 4 lexical access, for example. However, given the sample size of this pilot work, there are certain limitations to this type of strategy, which is recommended for future lines of research.

While the present results appear to be promising, there are several aspects or limitations that need to be highlighted. Firstly, a relationship between age and WAY2AGE scores, but not between age and MMSE scores, was found. This would indicate that, even if WAY2AGE seems sensitive to detect MCI, the voice tool is age-sensitive and not as robust as the traditional MMSE scale. Future lines of research should look more deeply into parameters that distinguish developmental changes from changes in cognitive impairment in the proposed bot. Of note, age and education differences are issues that have been worked on later, in the MMSE adaptations through suitable cut-offs [27, 28]. Additionally, conducting an extensive cognitive evaluation to definitively discern whether patients exhibit MCI would be highly recommended in future studies. This comprehensive assessment would enable researchers to gain a more accurate understanding of the cognitive abilities and potential impairments of the participants, allowing for a more precise classification and diagnosis of their cognitive conditions. Lastly, participants’ medication was not considered in the current research. Measuring medication is crucial for further research as it allows researchers to accurately assess whether any observed outcomes can be attributed to the variables of interest rather than other factors.

This study is a first step in the study of screening methods using WAY2AGE, more precisely, voice-based measures. At the applied level it is of interest for healthcare workers. Failure to identify dementia is costly to society in the long term. In this way, the MCI screening at an early progression stage is important for interventions to be effective, as therapy must be initiated before the onset of extensive brain tissue damage. AI based approaches could be an affordable opportunity. However more research that shed lights on how to approach AI to older adults is needed. As a systematic review indicates, it is necessary to understand the needs of older adults in order to design and develop new items to meet their needs [7].

## Data Availability

The datasets generated during the current study are available from the corresponding author on reasonable request.
