# Correction: A pilot screening for cognitive impairment through voice technology (WAY2AGE)

**DOI:** 10.1186/s40359-024-01555-6

**Published:** 2024-02-06

**Authors:** Carmen Moret-Tatay, Isabel Iborra-Marmolejo, María José Jorques-Infante, Gloria Bernabé-Valero, María José Beneyto-Arrojo, Tatiana Quarti Irigaray

**Affiliations:** 1 Faculty of Psychology, Valencia Catholic University Saint Vincent Martyr (UCV), Burjassot, Valencia, Spain; 2 Department of Psychology, Pontifical Catholic University of Rio Grande do Sul (PUCRS), Rio Grande do Sul, Brazil

**BMC Psychology (2023) 11:170**

10.1186/s40359-023-01212-4

Following publication of the original article [1], the authors brought the following concerning their use of the ‘MMSE’ instrument to the attention of the journal: We used an unauthorized version of the Spanish MMSE without permission from PAR. PAR have now granted retrospective permission. The MMSE is a copyrighted instrument and may not be used or reproduced in whole or in part, in any form or language, or by any means without written permission of PAR (www.parinc.com).

This is for the reader’s reference and does not affect the results of the article.

